# Autophagy induced by baicalin involves downregulation of CD147 in SMMC-7721 cells *in vitro*

**DOI:** 10.3892/or.2011.1599

**Published:** 2011-12-20

**Authors:** XIANJIAO ZHANG, XU TANG, HANQIANG LIU, LIANXIANG LI, QIAN HOU, JIANMIN GAO

**Affiliations:** 1 School of Medicine, Xi’an Jiaotong University, Xi’an; 2 Department of Pathology, Sichuan College of Traditional Chinese Medicine, Mianyang; 3 Department of Nutrition and Food Hygiene, The Fourth Military Medical University, Xi’an; 4 Department of Infection Control and Prevention, Shaanxi Provincial People’s Hospital, Xi’an; 5 Department of Traditional Chinese Medicine, Xijing Hospital, The Fourth Military Medical University, Xi’an; 6 School of Public Policy and Administration, Xi’an Jiaotong University, Xi’an, P.R. China

**Keywords:** baicalin, hepatocellular carcinoma cell, autophagy, apoptosis, CD147

## Abstract

Baicalin has been demonstrated to exert anticancer effects mainly through induction of tumor cell apoptosis and cell cycle arrest. However, the precise mechanisms underlying its anticancer role remain to be elucidated. In the present study, we investigated whether autophagy was involved in the anticancer activity of baicalin in the human hepatocellular carcinoma (HCC) cell line SMMC-7721 and the possible molecular mechanisms. Our data showed that the viability of SMMC-7721 cells was significantly inhibited by baicalin in a dose- and time-dependent manner. Alongside apoptosis, autophagy was also induced by baicalin dose- and time-dependently with the involvement of the autophagy-associated protein Beclin 1. Moreover, we demonstrated that cell death induced by baicalin was significantly inhibited by the apoptosis inhibitor z-DEVD-fmk or the autophagy inhibitor 3-MA, respectively. In addition, we found that CD147, a key molecule related both to apoptosis and autophagy, was markedly downregulated at the protein level in SMMC-7721 cells treated with baicalin. Collectively, this is the first study to suggest that baicalin induces autophagic cell death in SMMC-7721 cells, which involves the downregulation of CD147. Our study reveals a new mechanism for the anticancer effects of baicalin and puts forward a potential crucial role of CD147 in baicalin-induced cancer cell death.

## Introduction

Hepatocellular carcinoma (HCC) is one of the most prevalent tumor types worldwide, especially in Asian countries, and both incidence and mortality rates of HCC have increased in recent years (1). Hepatic resection and transplantation are potential curative treatments for HCC, but only about 20% of the patients are suitable candidates (2). For the treatment of unresectable HCC, chemotherapy represents an important tool in the clinical therapy of HCC. However, severe cytotoxicity induced by most of the commonly used anticancer drugs on normal cells necessitates the screening and development of some novel therapeutic reagents with relatively low side effects (3). Recently, traditional Chinese medicines have been recognized as a new source of anticancer drugs and new adjuvant therapy to enhance the efficacy of chemotherapy and to reduce the cancer chemotherapy-related side effects (4). In this regard, Chinese traditional herbs have been of particular interest, due to their relatively low toxicity as concluded from their extensive clinical usage in the past. Isolation and screening of the active components from the herbs possessing anticancer potential appears to be a promising way of discovering novel therapeutic compounds.

The Chinese herb *Scutellaria baicalensis* (SB) is a member of the Lamiaceae or mint family and is known as Chinese skullcap (common name: Huang-Qin in China) and as Japanese Ogon. SB has been widely used in traditional Chinese medicine with multi-properties, such as antitumor, anti-inflammatory, anti-hypertensive, anti-cardiovascular, antibacterial and antiviral (4). Diverse phytoestrogen-like substances mainly including baicalin, baicalein and wogonin have been isolated from SB. Accumulating studies have demonstrated that baicalin possibly was the key bioactive ingredient mediating the anticancer effect of SB (4). Baicalin exerts strong suppressive effects on many types of cancer cells and has been documented to be a promising anticancer candidate (4–7). However, the precise mechanisms underlying its anticancer effects remain to be elucidated.

Autophagy is a highly conservative intracellular process for degrading long-lived proteins and cytoplasmic organelles that consists of several sequential steps: sequestration, transport to lysosomes, degradation and utilization of degradation products (8). It is characterized by the appearance of double- and multi-membrane cytoplasmic vesicles that engulf cytoplasm and organelles, forming autophagosomes marked by microtubule-associated protein light chain 3 (LC3) (9). Many key molecules are involved in this biological process, especially Beclin 1, a mammalian homolog of yeast Atg6/Vps30 and an essential regulator that promotes autophagosome formation through mediating the localization of other autophagy proteins on the pre-autophagosomal membrane (10). Autophagy plays a wide variety of roles in physiological and pathological processes, such as starvation adaptation (11), embryonic development (12), cell survival and death (13), and tumor suppression (14). Recent publications have reported two seemingly opposite functions of autophagy in tumor progression (8,15). Based on the ability of autophagy to promote cell survival in response to metabolic stress, it has been suggested that autophagy may contribute to tumor development. On the other hand, autophagy also directly or indirectly induces autophagic cell death through excessive self-digestion and the activation of apoptosis and inhibits tumor progression (14). In this context, it is a novel anticancer strategy to induce autophagic cell death in cancer cells and this concept has been confirmed with several chemotherapy agents from traditional Chinese medicine such as berberine (16,17) and arsenic trioxide (18). However, it has never been investigated whether baicalin induces autophagy in cancer cells.

CD147, a glycosylated immunoglobulin superfamily transmembrane protein, is highly expressed in HCC cell lines and tumor tissues (19,20). Several studies *in vitro* have suggested that CD147 inhibits tumor cell apoptosis (21) and anoikis (22), promotes invasion and metastasis (19,23,24), enhances tumor angiogenesis (25), and conferrs chemoresistance to some drugs (22). These findings indicate that CD147 may serve as a key therapeutic target for HCC. Studies from our laboratory demonstrated that CD147 may play an important role in the inhibitory regulation of autophagy and autophagic cell death in HCC cells (17,26). However, whether CD147 also plays a role in mediating the anticancer effects of baicalin remains unclear.

In the present study, we sought to assess the effects of baicalin on tumor cell growth, autophagy induction and the possible molecular mechanisms underlying baicalin-induced cell death in SMMC-7721 cells *in vitro*. Our results demonstrate that baicalin induced both autophagic cell death and apoptosis with downregulation of CD147 expression in SMMC-7721 cells *in vitro*. To the best of our knowledge, this is the first study to investigate whether baicalin induces autophagy and the relationship between baicalin-induced cell death and CD147 expression.

## Materials and methods

### Materials

Baicalin was purchased from the China National Information Infrastructure for Reference Materials (Beijing, China) and dissolved in normal saline (NS) to 20 mg/ml, pH 7.2, then vortexed at room temperature for 10 min. This solution was centrifuged at 5,000 rpm for 10 min to remove any insoluble ingredients, then the supernatant was passed through a 0.22-μm pore-size filter (Millipore, Bedford, MA) for sterilization and diluted in RPMI-1640 medium at a final concentration of 1 mol/l (M) as a stock solution. The pEGFP-LC3 plasmid was kindly provided by Professor Xingchun Gou (Institute of Basic Medicine, Xi’an Medical University, Xi’an, China). All reagents were obtained from common commercial sources.

### Cell culture

SMMC-7721 cells were provided by the Institute of Cell and Biochemistry, Chinese Academy of Sciences (Shanghai, China) and cultured in RPMI-1640 medium supplemented with 10% heat-inactivated (56°C) fetal bovine serum (FBS) and 100 U/ml penicillin, 100 μg/ml streptomycin at 37°C in a humidified atmosphere with 5% CO_2_. To study baicalin-induced cell death, SMMC-7721 cells were cultured either with or without baicalin (treatment group or control group).

### MTT assay

Cell viability was determined after treatment with various concentrations of baicalin and different durations of exposure to baicalin by the MTT assay as previously described (27). In brief, SMMC-7721 cells were seeded at a density of 5×10^3^ cells/well in 96-well microplates in RPMI-1640 medium, and were cultured for 24 h. Then, the cells were treated with various concentrations (0, 10, 20, 40, 80, and 160 μM) of baicalin for different times (0, 24, 48 or 72 h). Subsequently, 10 μl of 5 mg/ml MTT (Sigma Chemical Co., USA) was added to each well for an additional 4-h incubation and the resulting formazan crystals were dissolved in 100 μl DMSO. Finally the optical density (OD) at 570 nm was measured using an automatic microplate reader (Immuno Mini NJ-2300; Inter Med, Tokyo, Japan). The percentage of cell viability inhibition was calculated according to the following formula: [(OD value of the control cells - OD value of the treated cells)/OD value of the control cells] × 100%. The viability of the control cells, from the untreated cultures, was defined as 100%.

### Autophagy assay with LC3 dots

SMMC-7721 cells were transfected with pEGFP-LC3 for 24 h and then cultured with different concentration of baicalin (0, 40, 80 or 160 μM). Exogenous enhanced GFP (EGFP)-fused microtubule-associated protein light chain 3 (EGFP-LC3) is a specific autophagy marker widely used in autophagy research (28). When autophagy is stimulated, the distribution pattern of GFP-LC3 changes from a diffuse cytoplasmic pattern to a punctate pattern that labels pre-autophagosomal and autophagosomal membranes (29). Autophagy was analyzed using an Olympus BX60 fluorescence microscope (Olympus, Tokyo, Japan). The percentage of cells with EGFP-LC3 punctate dots was determined as previously described (26). Briefly, a minimum of 100 cells from each sample was counted in three independent experiments. The percentage of cells showing EGFP-LC3 punctate dots was calculated by dividing the number of cells showing punctate dots by the number of cells counted.

### Transmission electron microscope (TEM) analysis

A TEM analysis was performed as previously described (26). Briefly, after culture in the absence or presence of 160 μM baicalin for 48 h, SMMC-7721 cells were fixed with 3% glutaraldehyde in 0.2 M phosphate buffer (pH 7.3) for 4 h at 4°C, then post-fixed with 1% osmium tetroxide and 0.5% tannic acid for 1 h at 4°C followed by washing three times with 0.1 M phosphate buffer (pH 7.3). Next, cells were dehydrated and embedded in Epon812 (Electron Microscopy Sciences, Fort Washington, PA, USA). Finally, sections were counterstained with uranyl acetate and lead citrate, and examined using a JEM-2000EX transmission Electron Microscope (Jeol Ltd., Tokyo, Japan).

### Immunofluorescence staining

SMMC-7721 cells were stained for Beclin 1 (Abcam Co., USA) by immunofluorescence followin the protocol as detailed previously with the following modifications (30). Briefly, cells on coverslips were fixed in 4% paraformaldehyde for 15 min and permeabilized with 0.1% Triton X-100 for 10 min, washed three times (PBS containing 0.01% Triton X-100 and 10% FBS), followed by incubation with the primary antibody (anti-human Beclin 1) overnight at 4°C, washed, and incubated with protein-blocking solution. Subsequently, cells were incubated for 1 h at room temperature with a secondary antibody (goat-anti mouse IgG) that was conjugated to fluorescein isothiocyanate (FITC, green fluorescence) (Molecular Probes Inc., Eugene, Oregon, USA) and then washed. Finally, images were captured using an Olympus BX60 (Olympus Optical Co., Ltd., Tokyo, Japan) fluorescence microscope.

### Western blot analysis

Beclin 1 and CD147 protein expression in SMMC-7721 cells was detected by Western blotting performed as previously described (25). Briefly, cells were washed twice with ice-cold PBS. Cell samples were lysed with RIPA buffer (Pierce Biotechnology, Inc., USA) for 45 min on ice and protein concentrations were measured using the BCA kit (Pierce Biotechnology, Inc.). Equal amounts of protein (10 μg) were separated by 10% SDS-PAGE and electrophoretically transferred to polyvinylidene difluoride membranes (Millipore, Bedford, MA) using a mini trans-blot apparatus (Bio-Rad Laboratories). Membranes were blocked with PBS-0.05% Tween-20 containing 5% non-fat dry milk for 1 h and incubated with monoclonal mouse anti-human Beclin 1 and CD147 or tubulin antibody (Palo Alto, CA, USA) for 2 h at room temperature. Membranes were then washed three times with PBS-0.05% Tween-20 and incubated with HRP-labeled goat anti-mouse antibody (Carlsbad, CA, USA) at a 1:10,000 dilution for 1 h. Blots were developed using an enhanced chemiluminescence kit (Pierce Biotechnology, Inc.). Each experiment was repeated at least three times.

### Trypan blue dye exclusion assay

Cell viability of SMMC-7721 cells was assessed by the trypan blue exclusion assay as previously described (31). Briefly, SMMC-7721 cells were pretreated with 0, 40, 80 or 160 μM baicalin for 48 h in the absence or presence of the autophagy specific inhibitor 3-MA (100 μM) (Sigma Chemical Co.) and/or the caspase-3 specific inhibitor Zyloxy-Asp-Glu-Val-Asp fluoromethyl ketone (z-DEVD-fmk) (100 nM) (Beyotime Institute of Biotechnology, China). Cells were washed with PBS, trypsinized, and collected by centrifugation, and then resuspended in 200 μl PBS. After mixing with 200 μl of 0.8% trypan blue, the cells were counted using a hemocytometer. The number of dead cells with disrupted membranes (blue cells) per 200 cells was counted in triplicates. Cell death was expressed as the mean percentage of blue cells/total cells.

### Statistical analysis

All statistical analyses were performed using the SPSS 16.0 statistical package for Microsoft Windows (SPSS, Chicago, IL). Statistical significance was determined using a Student’s t-test. All tests in this study were two-sided and P<0.05 was considered statistically significant.

## Results

### Antiproliferative effect of baicalin on SMMC-7721 cells

**Figure 1:**
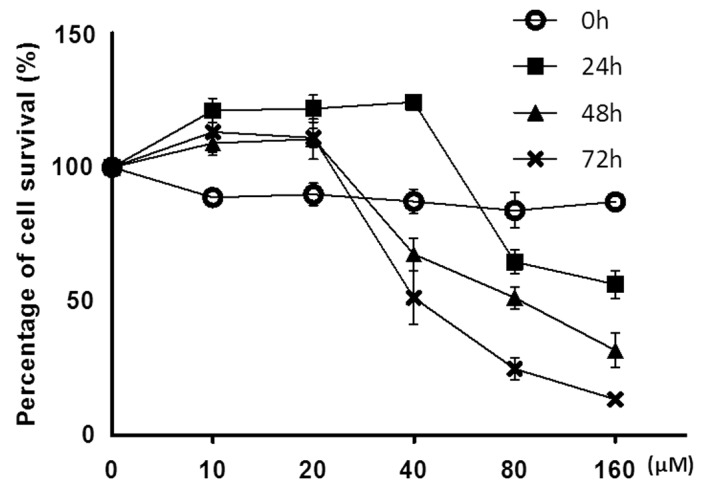
Baicalin inhibits SMMC-7721 cell proliferation *in vitro*. The viability of SMMC-7721 cells after baicalin treatment was analyzed using the MTT assay. The percentage of cell viability was calculated according to the following formula: [(OD value of the control cells - OD value of the treated cells)/OD value of the control cells] × 100%. The viability of the control cells, from the untreated cultures, was defined as 100%. Results are representative of three independent experiments. Error bars represent the standard deviation (SD). P-values were calculated using an unpaired Student’s t-test.

We first examined the effect of baicalin on the survival of SMMC-7721 cells using the MTT assay. Our results indicated that baicalin displayed strong inhibitory effect on the growth of SMMC-7721 cells in a dose and time-dependent manner (Fig. 1), and the IC_50_ value of baicalin was determined to be 40 μM for 72 h or 80 μM for 48 h (Fig. 1). Apparently, at a concentration as high as 160 μM, baicalin provoked the strongest inhibitory effect on SMMC-7721 cells, with morphological alterations characteristic of cell death observed under a light microscope (data not shown). However, at a low dose range (10 and 20 μM), baicalin failed to elicit a significant inhibitory effect on SMMC-7721 cells vitality. Thus, a baicalin concentration ≥40 μM was used for subsequent experiments.

### Autophagy induced by baicalin in SMMC-7721 cells

**Figure 2:**
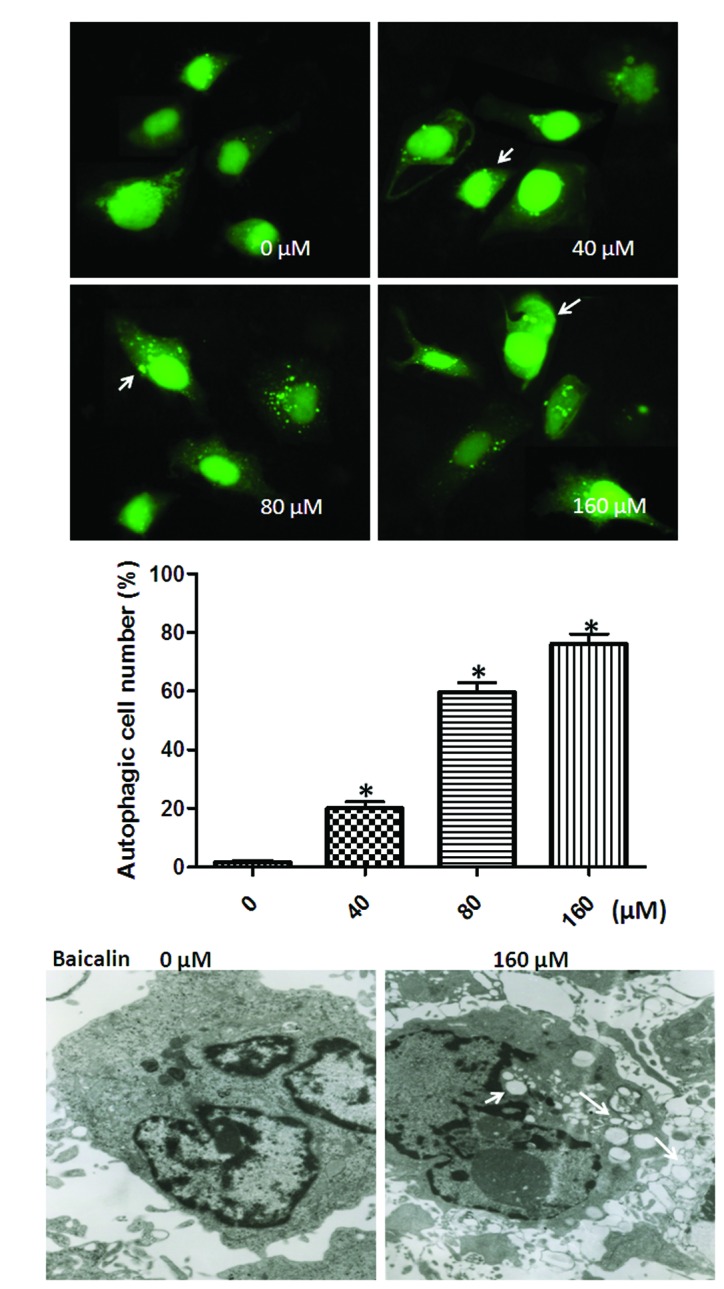
Baicalin induces autophagy in SMMC-7721 cells. SMMC-7721 cells were transfected with pEGFP-LC3, and then treated with the indicated concentrations of baicalin (0, 40, 80 and 160 μM) for 48 h. Cells were viewed under a fluorescence microscope. Results shown are representative of three independent experiments. (A) Representative micrographs showing autophagosomes (white arrows). (B) Mean percentage of cells with LC3 punctate dots from triplicate samples in the different treatment groups. Error bars represent ± SD. Statistical significance was determined using an unpaired Student’s t-test. ^*^P<0.01 vs. 0 μM. (C) SMMC-7721 cells were cultured with 0 or 160 μM baicalin for 48 h before harvesting and analysis by transmission electron microscopy. The cells from the control group (without baicalin treatment) showed typical normal cancerous structures, with large and round nuclei, uniform chromatin density and clear nucleoli. However, the cells treated with 160 μM baicalin for 48 h show signs of autophagy and apoptosis. White arrows indicate autophagic vesicles and white arrowheads indicate apoptosis.

To determine whether baicalin could induce autophagy, the SMMC-7721 cells were transfected with a plasmid expressing an pEGFP-LC3 fusion protein and then exposed to different concentrations of baicalin (40, 80 or 160 μM) for 48 h. The formation of EGFP-LC3 punctate dots is a widely used marker for autophagy, which can be easily monitored by a fluorescence microscope (32). As expected, in normal condition without baicalin treatment, EGFP-LC3 fluorescence was largely diffused throughout the cytoplasm with few dots denoting basal autophagosome formation. However, the number of EGFP-LC3 dots rapidly increased within 24-h exposure to baicalin (40, 80 or 160 μM), indicating that autophagy was induced. The percentage of autophagic cells with more than three GFP-LC3 puncta (GFP-LC3 positive) was 20.4% at 40 μM, 61.2% at 80 μM, and 79.7% at 160 μM baicalin, respectively, indicating that autophagy was induced by baicalin in a dose-dependent manner (Fig. 2A and B).

To further confirm the effect of baicalin on autophagy, we evaluated the level of baicalin-induced autophagy in SMMC-7721 cells using a TEM method, which is currently the standard method for monitoring autophagy. As shown in Fig. 2C, autophagic vacuoles (white arrows) were observed in SMMC-7721 cells treated with 160 μM baicalin. The results suggest that a significantly higher level of autophagy occurred in SMMC-7721 cells treated with 160 μM baicalin compared with that in the control (0 μM baicalin). Moreover, we observed that cell nuclei had collapsed and disintegrated in cancer cells treated with baicalin (white arrowheads) (Fig. 2C), which indicated that baicalin also induced apoptosis and was in line with results of previous studies (33,34). Taken together, all these results clearly demonstrated that baicalin induced autophagy as well as apoptosis in SMMC-7721 cells.

### Baicalin upregulates autophagy-related protein Beclin 1 expression

**Figure 3:**
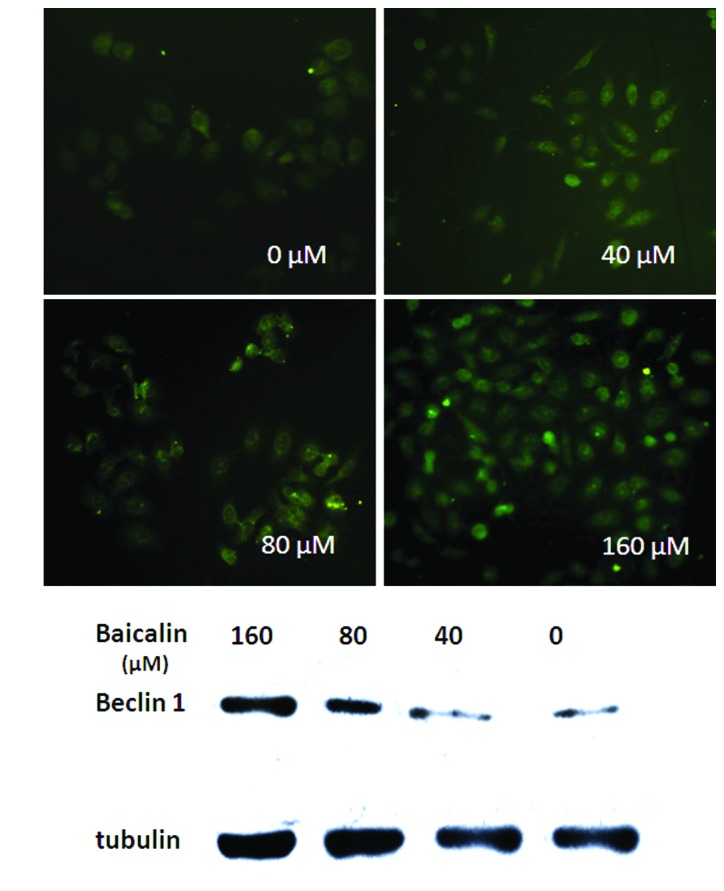
Baicalin upregulates Beclin 1 protein levels in SMMC-7721 cells. (A) Immunofluorescence staining and (B) Western blot analyses for Beclin 1 protein levels in SMMC-7721 cells treated with baicalin (0, 40, 80 or 160 μM) for 48 h.

To investigate the mechanisms by which baicalin induces autophagy, we detected the protein levels of Beclin 1, the key regulator of autophagy which has already been shown to be essential for the occurrence of autophagy (30). Following the treatment on SMMC-7721 cells with different concentrations of baicalin (40, 80 or 160 μM) for 48 h, we performed the immunofluorescence staining and Western blot analysis to evaluate the expression level of Beclin 1. Immunofluorescence staining showed that Beclin 1 abundance was remarkably higher in SMMC-7721 cells treated with baicalin compared to control in a dose-dependent manner (Fig. 3A). A similar result was obtained by Western blot analysis (Fig. 3B). These findings suggest that autophagy induced by baicalin in SMMC-7721 cells is, at least in part, Beclin 1-dependent.

### Baicalin induces both autophagic cell death and apoptosis

**Figure 4:**
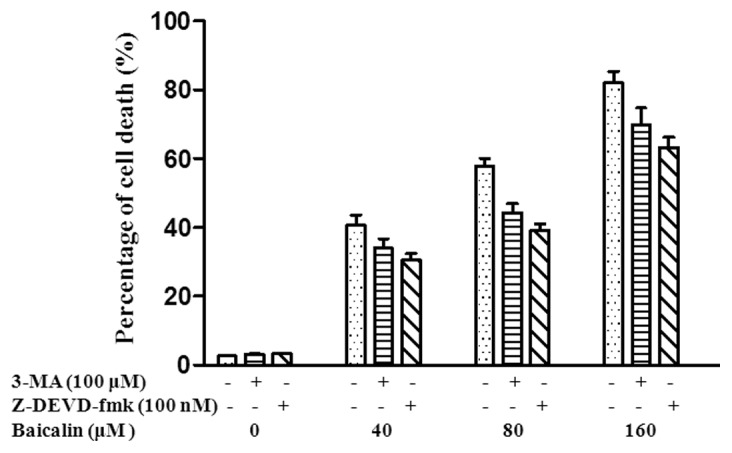
Baicalin induces autophagic cell death and apoptosis in SMMC-7721 cells. SMMC-7721 cells were pretreated with various concentrations of baicalin (0, 40, 80 and 160 μM) in the absence or presence of 3-methyladenine (3-MA) and/or z-DEVD-fmk. The results show that both 3-MA and z-DEVD-fmk partly inhibited cell death. Data were presented as mean ± SD of three separate experiments, each of which were performed in triplicate.

Autophagy in cancer cells has been demonstrated to play a dual role in cell survival and cell death (15). To further determine the functional role of autophagy induced by baicalin in cancer cell survival or death and the relationship between autophagy and apoptosis, we investigated baicalin-induced cell death in SMMC-7721 cells using a Trypan blue exclusion assay. 3-Methyladenine (3-MA), a known inhibitor of autophagy, was used to inhibit autophagy and prevent autophagic cell death induced alone (35). z-DEVD-fmk, a caspase-3-specific inhibitor of apoptosis, inhibits only apoptosis with no other types of cell death (36). SMMC-7721 cells were treated with 3-MA (100 μM), z-DEVD-fmk (100 nM) or 3-MA (0 μM) + z-DEVD-fmk (0 nM), and then cells were treated with 40, 80 and 160 μM baicalin. The percentage of cell death was 33.9, 30.5 and 40.6%, respectively in SMMC-7721 cells treated with 40 μM baicalin. The percentage of cell death was 44.3, 39.1 and 57.7%, respectively in SMMC-7721 cells treated with 80 μM baicalin and 69.9, 63.3 and 82.1%, respectively in SMMC-7721 cells treated with 160 μM baicalin (Fig. 4). Collectively, our results demonstrate that cell death is inhibited by both 3-MA and z-DEVD-fmk, respectively, which further suggests that baicalin-induced cell death including both autophagic cell death and apoptosis.

### Identification of cell death-related genes that regulate autophagy and apoptosis

**Figure 5:**
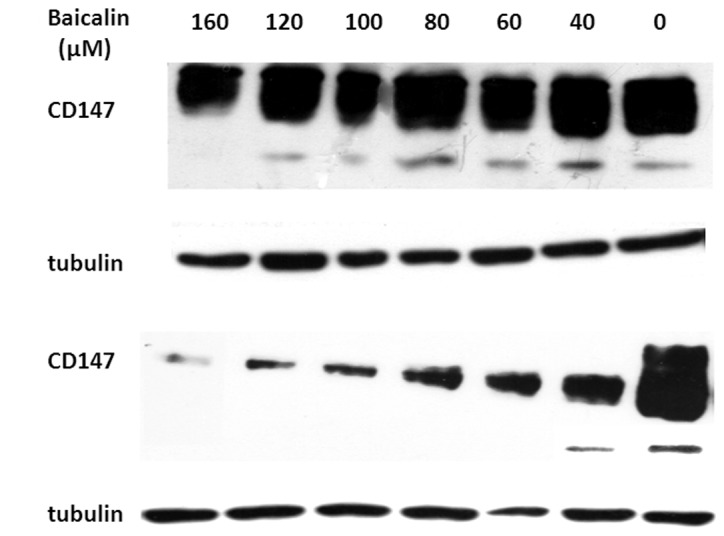
Baicalin downregulated CD147 expression. Western blot analysis for CD147 protein levels in SMMC-7721 cells treated with the different concentrations of baicalin (0, 40, 60, 80, 100, 120 and 160 μM) for (A) 24 h or 48 h (B). Tubulin was used as a protein loading control in the Western blot assay.

Recent studies have uncovered significant interactions between autophagic and apoptotic signaling pathways (37,38). It has been reported that autophagy and apoptosis may be linked to each other and occur simultaneously or sequentially in cancer cells (39). To better understand the relationship between autophagy and apoptosis in SMMC-7721 cells treated with baicalin, we investigated whether some known cell death genes related both to autophagy and apoptosis, were involved in baicalin-induced cell death. Previous studies have suggested that CD147 inhibited apoptosis (21) and starvation-induced autophagic cell death (26), and was related with cell cycle arrest in SMMC-7721 cells (32). We thus investigated CD147 expression levels in SMMC-7721 cells treated with different concentrations of baicalin (40, 60, 80, 100, 120 or 160 μM). Western blotting results showed that CD147 expression level was significantly downregulated in SMMC-7721 cells treated with different concentrations of baicalin (40, 60, 80, 100, 120 and 160 μM) for 24 h (Fig. 5A) and 48 h (Fig. 5B) compared to the controls (cells without baicalin treatment) in a dose-dependent manner at protein levels. The data suggest that downregulation of CD147 expression may be involved in both baicalin-induced autophagy and apoptosis.

## Discussion

Currently, chemotherapy using plant-derived anticancer drugs such as paclitaxel (40), vinorelbine (41), or vincristine (42) has been proven to be effective and safe in many clinical settings. It was shown that these products enhance cell growth inhibition, induce apoptosis or cell cycle arrest in several cancer cell lines and are highly effective and safe in clinical trials. Noticeably, about 75% of plant-derived drugs used today in the clinic originate from traditional medicines (43). Our previous result showed that berberine isolated from the Chinese herb *Scutellaria baicalensis* (SB) Georgi coule induced both apoptosis and autophagy in the HCC lines HepG2 and SMMC-7721 (17).

In the present study, we investigated the anticancer effects of baicalin, another ingredient isolated from SB, on HCC SMMC-7721 cells *in vitro*. Our results showed that baicalin exerted a significant antiproliferative effect on SMMC-7721 cells, in line with previous studies which demonstrated the antiproliferative effect of baicalin in other cancer cell lines (PC-3, DU145, LNCaP, MCF-7, HL-60, and NB4) (33,34,44,45).

Our next attempt was to find out the underlying mechanisms of baicalin on SMMC-7721 cell proliferation inhibition. Previous studies indicated that baicalin induced apoptosis and cell cycle arrest in many types of cancers which contributed to cell growth inhibition (33,34,44,45). However, whether baicalin induced other types of cell death such as autophagic cell death in cancer cells remains unknown. The present study is the first to provide compelling experimental evidence that baicalin induced autophagy in SMMC-7721 cells. This finding sheds new light on the pharmacological function of baicalin. Cancer is one of the first diseases genetically linked to autophagy malfunction (8,46). The ATG gene Beclin 1, as an important autophagy-associated signaling molecule, is mono-allelically deleted in 40–75% of human malignancies such as liver, lung, breast, ovarian, and prostate cancer (14). The restoration of Beclin 1 expression inhibits tumor cell proliferation and tumor growth (47). Moreover, upregulation of Beclin 1 expression mediates anticancer agent-induced autophagy (48). In the present study, we demonstrated that the expression of Beclin 1 was upregulated by baicalin in a dose-dependent fashion and positively associated with the level of autophagy, indicating that upregulation of Beclin 1 expression may be one of the major mechanisms of baicalin-induced autophagy. The detailed mechanisms underlying the inter-relationships between autophagy, cell survival and cell death are largely unknown. Many studies showed that autophagy augmentation may be effective in preventing tumour formation and progression, whereas autophagy inhibition may be helpful in promoting tumour regression (8,46). Currently, many studies have summarized the essential connections between defects in autophagy regulation or execution and cancer, and suggested that autophagy was a true tumour suppressor pathway (49). Several chemotherapy agents such as berberine (16,17) and arsenic trioxide (18) have been demonstrated to induce autophagic cell death in cancer cells and thus exert anticancer activity. Therefore, in the present study we determined the functional role of autophagy induced by baicalin in SMMC-7721 cell survival or death. We further investigated whether baicalin could induce autophagic death in SMMC-7721 cells, defining a novel role of baicalin as an antitumor agent. Our results indicated that alongside apoptosis, baicalin also induced autophagic cell death in SMMC-7721 cells. These results suggest that induction of autophagy, as well as apoptosis, by baicalin may represent a novel mechanism by which baicalin inhibits tumor cell growth and modulates tumor progression.

Apoptosis (Type I) and autophagic cell death (Type II) are the two types of programmed cell death, whereas the boundary between Type I and II has never been completely clear and perhaps does not exist, and different types overlap. Autophagy and apoptosis have been reported to be linked to each other and occur simultaneously or sequentially in cancer cells (39). Recently, several laboratories have reported that molecules previously defined as intermediaries in the activation of apoptosis also function as intermediaries in the activation of autophagy (37,50), suggesting these molecules may be involved in the modulation of both apoptosis and autophagy simultaneously. Targeting these molecules can be more effective than to target molecules modulating apoptosis or autophagy alone in cancer treatment. Thus, characterization of molecules that involve in the modulation of both apoptosis and autophagy has been actively pursued (51).

CD147 is the most commonly highly expressed gene in human cancers, mainly functions as a cellular adhesion molecule inducing the secretion of matrix metalloproteinases (MMPs) (19). Increased MMP expression was associated with tumor invasion and metastasis (19). Aside from stimulating MMPs production, CD147 also stimulates the production of vascular endothelial growth factor (VEGF) which serves as a major regulator of the angiogenic process in tumor formation (52). It has been reported that CD147 was associated with the cell cycle, inhibited cancer cell apoptosis (21) and anoikis (22). Recent studies have further addressed that CD147 inhibited autophagy in SMMC-7721 cells with an involvement of Beclin 1 downregulation (26). These findings open the possibility that CD147 may be an important modulator involved in both apoptosis and autophagy. Given the fact observed in our present study that cell death induced by baicalin in SMMC-7721 cells included both apoptosis and autophagy. We evaluated the difference of the CD147 expression levels in SMMC-7721 cells treated with different concentrations of baicalin. As expected, the results indicated that CD147 expression levels were markedly downregulated by baicalin accompanied with the occurrence of apoptosis and autophagy. Based on these results, it is reasonable to hypothesize that baicalin-induced both apoptosis and autophagy in SMMC-7721 cells, at least in part, by downregulating CD147 expression and CD147 may serve as an important modulator of cross-talk between apoptosis and autophagy. However, further experiments are needed to verify the mechanisms by which baicalin regulates the expression of the CD147 and induce the cell death including apoptosis and autophagy.

In conclusion, our results show for the first time that baicalin induces autophagic cell death as well as apoptosis in SMMC-7721 cells, at least in part, through a pathway involving downregulation of CD147. These results suggest a novel mechanism underlying the pharmacological effects of baicalin and provide new insights into the function of CD147 during tumor progression.
